# Numerical Analysis of an Autonomous Emergency Braking System for Rear-End Collisions of Electric Bicycles

**DOI:** 10.3390/s24010137

**Published:** 2023-12-26

**Authors:** Ying Zhao, Haijun Li, Yan Huang, Junyu Hang

**Affiliations:** 1 School of Traffic and Transportation, Lanzhou Jiaotong University, Lanzhou 730070, China; 17791327079@163.com (Y.Z.); yanhuang@mail.lzjtu.cn (Y.H.); 2 Key Laboratory of Railway Industry on Plateau Railway Transportation Intelligent Management and Control, Lanzhou 730070, China; 3 MOT Key Laboratory of Transport Industry of Big Data Application Technologies for Comprehensive Transport, School of Traffic and Transportation, Beijing Jiaotong University, Beijing 100044, China; 18114033@bjtu.edu.cn

**Keywords:** autonomous emergency braking system, electric bicycle, rear-end collision, e-bicycle following model, safety surrogate measure

## Abstract

The rapid growth in the number of electric bicycles (e-bicycles) has greatly improved daily commuting for residents, but it has also increased traffic collisions involving e-bicycles. This study aims to develop an autonomous emergency braking (AEB) system for e-bicycles to reduce rear-end collisions. A framework for the AEB system composed of the risk recognition function and collision avoidance function was designed, and an e-bicycle following model was established. Then, numerical simulations were conducted in multiple scenarios to evaluate the effectiveness of the AEB system under different riding conditions. The results showed that the probability and severity of rear-end collisions involving e-bicycles significantly decreased with the application of the AEB system, and the number of rear-end collisions resulted in a 68.0% reduction. To more effectively prevent rear-end collisions, a low control delay (delay time) and suitable risk judgment criteria (TTC threshold) for the AEB system were required. The study findings suggested that when a delay time was less than or equal to 0.1 s and the TTC threshold was set at 2 s, rear-end collisions could be more effectively prevented while minimizing false alarms in the AEB system. Additionally, as the deceleration rate increased from 1.5 m/s^2^ to 4.5 m/s^2^, the probability and average severity of rear-end collisions also increased by 196.5% and 42.9%, respectively. This study can provide theoretical implications for the design of the AEB system for e-bicycles. The established e-bicycle following model serves as a reference for the microscopic simulation of e-bicycles.

## 1. Introduction

Electric bicycles (E-bicycles) have become a common mode of commuting for some residents in China’s large and medium-sized cities due to their convenience, flexibility, and ability to bypass traffic congestion. In China, the number of e-bicycles sold in 2016 reached 3 million, with over 200 million e-bicycles on the road [1]. The rapid growth of e-bicycles has caused a series of safety problems. The statistics for China revealed that e-bicycle riders were involved in approximately 56,200 traffic accidents from 2013 to 2017, resulting in 8431 fatalities and 63,400 injuries [2]. According to hospitalization records [3], the head is the most frequently injured area for e-bicycle riders, accounting for approximately 46.4%. More than one-third of injured e-bicycle riders have experienced traumatic brain injuries. Fractures accounted for 51.1% of the nature of the injury, followed by superficial injuries accounting for 42.1%. Therefore, it is crucial to implement effective measures to reduce collisions involving e-bicycles and mitigate their severity.

To improve the traffic safety of e-bicycle riders, extensive studies have been conducted to identify characteristics of hazardous riding behavior [4,5,6,7,8,9]. Researchers have found that hazardous riding behaviors among e-bicycles, such as occupying motor vehicle lanes, manned riding, over-speeding, riding against traffic, red-light running, and using mobile phones while riding, are mainly caused by human factors [4,5,6,7]. In addition, non-human factors like weather conditions and road conditions [8,9,10] can also contribute to hazardous riding behaviors. To reduce hazardous riding behaviors and prevent collisions involving e-bicycles, various safety measures have been proposed, including strengthening traffic management and rider safety education [4,6], improving relevant laws and regulations [11,12,13], and further enhancing the riding environment for e-bicycles [14].

Although researchers have proposed numerous interference or preventive measures, most of these are policy-oriented and have limited effectiveness in reducing collisions involving e-bicycles without the application of an active safety system. The active safety system is commonly used in vehicles and involves utilizing the vehicle’s monitoring and control functions to prevent and reduce collisions. Previous studies have demonstrated the effectiveness of active safety systems [15,16,17,18,19,20,21]. For instance, Cicchino [15] discovered that the risk of serious or fatal rear-end collisions for trucks equipped with active safety systems decreased by 76%. Active safety systems necessitate the use of sensors like millimeter-wave radar, and with the recent decrease in the price of millimeter-wave radar in the past two years, it is gradually becoming more feasible to establish active safety systems on e-bicycles in terms of cost.

Taking into account that rear-end collisions are the most common type of traffic collisions [22], this study prioritized the prevention of rear-end collisions involving e-bicycles. The key to avoiding a rear-end collision is to control the speed of the vehicle and the following distance. In addition to conventional traffic signs and markings, active safety systems are commonly used to prevent rear-end collisions [15,20,21]. Therefore, an autonomous emergency braking system (AEB) was introduced based on millimeter-wave radar, and an e-bicycle following model was established in this study. Numerical simulation experiments were then executed using this model in various scenarios, and the effectiveness of AEB was evaluated based on the experimental results. Specifically, the objectives of this study are:

(1) To propose an AEB system for preventing rear-end collisions involving e-bicycles and verify its effectiveness in safety improvement.
(2) To establish an e-bicycle following model to accurately simulate the riding trajectory of e-bicycles.
(3) To examine the impact of delay time and the time-to-collision (TTC) threshold on the safety improvement effect of the AEB system and investigate the influence of deceleration conditions on the risk of rear-end collisions.

The rest of this study is organized as follows: In Section 2, the literature review is introduced. Section 3 outlines the framework of the AEB system and presents an e-bicycle following the model. In Section 4, the results of different evaluation indicators are systematically demonstrated. Section 5 reports the interpretations and discussions of the results. Finally, conclusions regarding the implications of the proposed AEB are given in Section 6.

## 2. Literature Review

### 2.1. Hazardous Riding Behavior

Numerous studies have been conducted on the hazardous riding behaviors associated with e-bicycles. The human factor is recognized as the most crucial factor that leads to hazardous riding behaviors involving e-bicycles [23]. Liu and Chen [6] argued that college students had a tendency to make phone calls while riding e-bicycles, and due to a lack of professional training, collisions involving riders were expected to occur. Based on the analysis of video data collected from 152 traffic collisions involving e-bicycles in China, Wang et al. [4] found that, in comparison to human factors, environmental factors had a less direct impact on the severity of collisions. Moreover, consistently occupying motor vehicle lanes was identified as a significant contributing factor to serious collisions. Yao and Wu [7] confirmed, based on self-reported questionnaire survey data, that male riders were more likely to be involved in at-fault collisions. The study also indicated that riders with stronger positive attitudes and more concern about collision risk were less likely to engage in hazardous riding behaviors. Following four days of on-site observations, Zhao et al. [5] discovered that over-speeding, manned riding, and riding against traffic were the primary hazardous riding behaviors displayed by e-bicycle riders.

Besides human factors, other factors such as weather conditions and road conditions are also associated with the hazardous riding behaviors of e-bicycles [8,9,10]. Zhang et al. [8] found that on rainy days, the rate of traffic violations by e-bicycles is higher compared to sunny and cloudy days. The traffic density on the road was also observed to significantly affect the hazardous riding behavior of e-bicycles occupying motor vehicle lanes. The use of sunshields, a device that blocks out the sun, could effectively reduce the incidence of e-bicycle riders running red lights at intersections, with a greater impact on sunny days than on cloudy days [9]. Du et al. [10] discovered that e-bicycle riders are more likely to wear helmets for safety while riding on rainy days compared to sunny and cloudy days.

### 2.2. Prevention of E-Bicycle Traffic Collisions

Researchers have proposed various relevant measures to prevent the occurrence of traffic collisions involving e-bicycles. From the perspective of strengthening traffic management and rider safety education, Wang et al. [4] believed that effective educational and intervention programs were needed to prevent high-risk riding behaviors among riders and improve the traffic environment. Liu and Chen [3] concluded that offering psychological counseling to college students can effectively cut down on hazardous riding behaviors. When deemed necessary, schools or traffic police may impose traffic fines as a measure. In terms of improving relevant laws and regulations, Carole et al. [13] highlighted the need for the creation of preventative guidelines or prospective guidelines to prevent traffic collisions involving e-bicycles. Truong et al. [11] put forward the suggestion that existing legislation should be enforced and integrated with comprehensive educational and publicity programs to effectively reduce the potential casualties related to using mobile phones while riding an e-bicycle. Wu et al. [12] suggested establishing policies that would promote the registration rate of e-bicycle licenses, thereby encouraging riders to ride legally. In the field of enhancing the riding environment for e-bicycles, Xu et al. [14] asserted that enhancing traffic facilities, improving traffic management measures, and providing safety education to riders were crucial measures in mitigating the collision risks associated with electric bicycles at signalized intersections.

Overall, the measures proposed by researchers to prevent traffic collisions involving e-bicycles are mostly policy-oriented, aimed at raising riders’ awareness of traffic safety and reducing traffic collisions caused by human factors through strengthening traffic management, safety education, and law enforcement.

### 2.3. The Effect of the Active Safety System

Active safety systems are widely recognized for their effectiveness in enhancing traffic safety and reducing the risk of collisions in various traffic scenarios. Wu et al. [21] found that active safety systems could help decrease drivers’ reaction time and lower the probability of rear-end collisions under fog conditions. Duan et al. [24] proposed an intelligent in-vehicle audio warning system specifically for merging behaviors in the work zone and demonstrated that the implementation of audio warnings can effectively reduce merging risks. Yan et al. [25] developed a collision warning system for red-light-running events at intersections and confirmed that the use of the collision warning system can effectively reduce the occurrence of red-light-running collisions. Additionally, the results also suggested that issuing warnings 4.0 s or 4.5 s in advance may be the proper warning time. Yang et al. [26] designed an in-vehicle audio warning system for grade crossings, and the results showed that the system had a significant positive impact on driver performance by reducing driving speed and promoting drivers to apply smooth braking.

To provide a comprehensive evaluation of active safety systems, some studies have also sought to investigate whether these systems lead to unwanted effects on behavior. Reinmueller et al. [18] investigated the adaptation of adverse behaviors in adaptive forward collision warning systems. The findings revealed that the use of adaptive systems, as compared to non-adaptive systems, led to a significant reduction in the drivers’ minimum time headway and TTC values while driving. Zhu et al. [19] examined the impact of forward collision warning systems on car-following behavior and found that forward collision warning systems equipped with headway monitoring functions can enhance traffic efficiency and stability without compromising safety.

### 2.4. The Effects of the TTC Value and System Delay

Time-to-collision (TTC) is a crucial measure in different warning systems and is commonly used to trigger warnings. Huang and Chao [27] proposed a lateral impact warning system-based two-indices TTC decision algorithm and introduced the grey prediction theory to estimate TTC to ensure drivers gain additional reaction time. Sun et al. [28] adopted the TTC and the relative distance as the warning indicators in the lane change warning system and gave warning thresholds in different stages. Wang et al. [29] designed a cooperative collision warning system based on TTC warning for intelligent vehicles, and the collision warning threshold was set to 3.0 s. Guillen and Gohl [30] tested the forward collision warning system that is based on the comfort boundary (CB) model, and the results demonstrated that drivers had a higher acceptance of the system with the warning outside the CB (TTC = 1.7 s). Considering the effectiveness and common use of TTC values in warning systems, the AEB system in this study also employs the TTC threshold as a risk judgment criterion.

Previous studies have extensively investigated the impact of system delay in various control systems. Xiang et al. [31] established a robust rear-end collision warning model based on dedicated short-range communication, considering system delay (transmission delay and information delay), and the system provided emergency warnings with improved performance. Lee et al. [32] developed a real-time forward collision warning system in a cloud-based communication environment and used an advanced feed-forward neural network to mitigate the impact of the communication delay. Ruan et al. [33] examined the impact of multiple time delays on the impact of heterogeneous traffic flow and found that time delays have a negative effect on the linear stability of heterogeneous traffic flow. An and Jung [34] proposed a cooperative lane change protocol considering communication delay for connected and automated vehicles, and the improved protocol was verified by implementing a longitudinal and lateral controller. Given the potential adverse consequences of system delay, it is crucial to verify the impact of different delay time conditions on system effectiveness when designing an AEB system.

### 2.5. Research Gap and Contributions of This Study

Previous studies have predominantly focused on identifying the characteristics of hazardous riding behaviors and summarizing the various types of such behaviors and their contributing factors. Researchers have proposed various preventive measures based on these characteristics, but most of them are policy-oriented and pose challenges in terms of effective enforcement. Additionally, active safety systems have been extensively proven to effectively and directly decrease collision risks. Hence, establishing a stable and low-cost active safety system on e-bicycles is worthwhile to effectively reduce the occurrence of collisions.

In response to the research gap, this study developed an AEB system for e-bicycles and proposed an e-bicycle following the model. A series of risk assessment indicators were selected, and numerical simulations were conducted in multiple scenarios to evaluate the system’s performance. Based on the experimental results, the study verified the effectiveness of the AEB system and assessed the impact of system delay time, system TTC threshold, and deceleration conditions on the risk of rear-end collisions. This study contributes to a systematic understanding of the changes in collision risk of e-bicycles equipped with an active safety system. The findings provide valuable theoretical references for the development of the AEB system for e-bicycles.

## 3. Methodology

**Figure 1:**
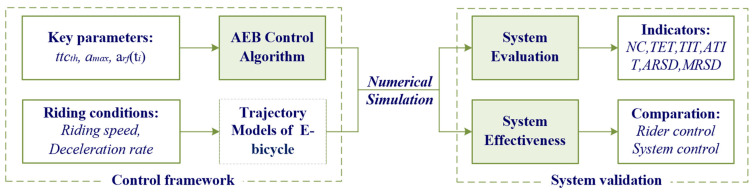
The research framework of this study.

The research framework of this study is shown in Figure 1. The framework of the AEB system was designed considering different key parameters, such as $ttc_{th}$, $a_{\max}$, $a_{rf}(t_{i})$. To obtain the riding trajectory data, the e-bicycle following model was proposed to simulate the e-bicycle dynamic data under various riding conditions. Combining the AEB control algorithm with the e-bicycle following model, a numerical simulation method was adopted to evaluate the effectiveness of the system. Based on the simulation results, the number of rear-end collisions and a series of improved safety surrogate measures were assessed under various riding conditions.

### 3.1. Framework Design of the Autonomous Emergency Braking (AEB) System

**Figure 2:**
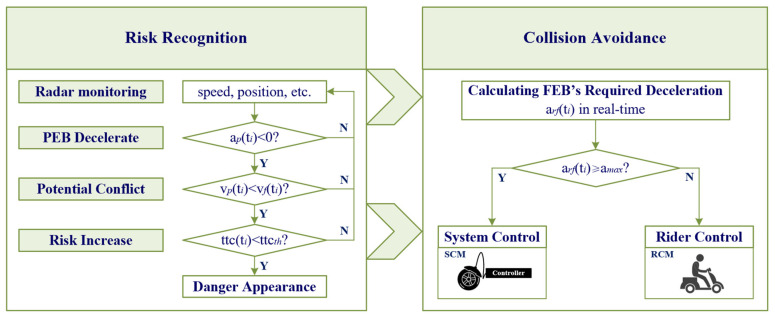
The process of the AEB system.

The AEB system proposed in this study consists of a risk recognition function and a collision avoidance function in an e-bicycle in the following situation, and the process flow is shown in Figure 2. During the collision avoidance process, which starts with the preceding e-bicycle’s (PEB’s) emergency deceleration and ends as the following e-bicycle (FEB) completes deceleration, the system monitors the speed and the distance headway of the two e-bicycles at a frequency of 10 Hz.

The system continuously monitors the positions and speeds of the preceding and following e-bicycles in real-time. When the PEB starts to decelerate, if the speed of the FEB is greater than that of the PEB, the system determines that a potential conflict appears. When potential conflicts arise, TTC between the two e-bicycles is monitored as the risk judgment criteria for the system. By definition, a smaller value of TTC has a higher collision risk, and vice versa. Therefore, choosing an appropriate TTC threshold is crucial to distinguishing between safe and hazardous zones. A larger TTC threshold allows for early detection of collision risks but may result in false alarms. On the other hand, a smaller TTC threshold provides accurate identification of collision risks but reduces system intervention ability. In the research on the warning systems in vehicles, commonly used TTC thresholds range from 1 to 3 s [35,36,37]. This study examined the effects of different TTC thresholds (i.e., 1 s, 2 s, and 3 s) on the effectiveness of the AEB.

When the actual TTC between the two e-bicycles falls below the TTC threshold, the system enters the collision avoidance stage. The system needs to calculate the required deceleration rate ($a_{rf}(t_{i})$) for the FEB in real-time to avoid rear-end collisions. If the $a_{rf}(t_{i})$ is equal to or greater than the maximum deceleration rate ($a_{\max}$) that the AEB system can provide, the AEB immediately enters the system control mode and activates the collision avoidance function with a deceleration of $a_{\max}$. Otherwise, the rider still maintains control over the e-bicycle. The specific formula for calculating $a_{rf}(t_{i})$ is shown in the next section.

### 3.2. E-Bicycle following Model

**Figure 3:**
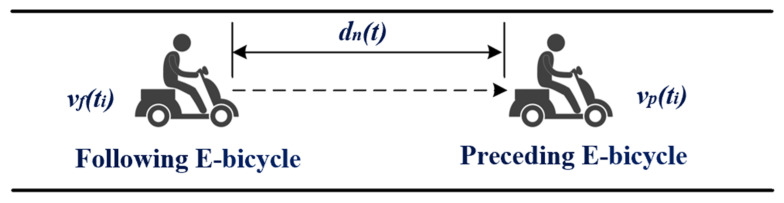
The relationship between the FEB and the PEB.

The intelligent driver model (IDM) is a classical model in the field of car-following models, and it is still frequently improved to adapt to various car-following scenarios [38,39,40]. Huang et al. developed the time variability of the time headway in the IDM model [38], and Jiang et al. proposed stochastic factors to change the unique relationship between speed and distance headway [39]. In this study, based on the formulation of the IDM and introducing the above characteristics, an e-bicycle model was proposed. Figure 3. illustrates the relationship between the PEB and the FEB. The accelerations of the PEB and FEB are expressed as follows:

(1) $$a_{p}(t_{i})=a_{e}\left[1-{\left(\frac{v_{p}(t_{i})}{v_{\max}}\right)}^{\theta }\right]+\xi$$

(2) $$a_{f}(t_{i})=a_{e}\left[2-{\left(\frac{v_{f}(t_{i})}{v_{\max}}\right)}^{\theta }-{\left(\frac{d_{en}(t_{i})}{d_{n}(t_{i})}\right)}^{2}\right]+\xi$$

(3) $$d_{n}(t_{i})=d_{p}(t_{i})-d_{f}(t_{i})$$

(4) $$d_{en}(t_{i})=\left\{ \begin{array}{l} D_{1}+(D_{2}-D_{1})\cdot r_{2},\;if\;r_{1}>(1-p_{1})\\ d_{en}(t_{i-1}),\;otherwise \end{array}\right.$$

Here, $a_{p}(t_{i})$ represents the acceleration of the PEB at the time $t_{i}$, $a_{e}$ represents the desired maximum acceleration, θ represents acceleration coefficient, $v_{p}(t_{i})$ represents the speed of the PEB at the time $t_{i}$, $v_{\max}$ represents the desired maximum speed, ξ represents the stochastic factors in e-bicycle following behavior, which is simply assumed to be uniformly distributed random number between $-\xi_{1}$ and $\xi_{1}$, $a_{f}(t_{i})$ represents the acceleration of FEB at the time $t_{i}$, $v_{f}(t_{i})$ represents the speed of the FEB at the time $t_{i}$, $d_{en}(t_{i})$ represents the desired distance headway at the time $t_{i}$, $d_{n}(t_{i})$ represents the distance headway between the FEB and PEB at the time $t_{i}$, $d_{p}(t_{i})$ represents the riding distance of the PEB at the time $t_{i}$, $d_{f}(t_{i})$ represents the riding distance of the FEB at the time $t_{i}$, $D_{1}$ and $D_{2}$ represent fixed distance headway, $r_{1}$ and $r_{2}$ are uniformly distributed random numbers between 0 and 1, $p_{1}$ represents a fixed probability.

Based on the $a_{p}(t_{i})$ and $a_{f}(t_{i})$, the speed and riding distance of the e-bicycles are updated. The specific mathematical expressions are as follows:

(5) $$v_{p}(t_{i})=v_{p}(t_{i-1})+\Delta t\cdot a_{p}(t_{i})$$

(6) $$v_{f}(t_{i})=v_{f}(t_{i-1})+\Delta t\cdot a_{f}(t_{i})$$

(7) $$d_{p}(t_{i})=d_{p}(t_{i-1})+v_{p}(t_{i-1})\cdot \Delta t+a_{p}(t_{i})\cdot {\left(\Delta t\right)}^{2}/2$$

(8) $$d_{f}(t_{i})=d_{f}(t_{i-1})+v_{f}(t_{i-1})\cdot \Delta t+a_{f}(t_{i})\cdot {\left(\Delta t\right)}^{2}/2$$

Here, Δt represents the length of a time step.

After calculating the speed and riding distance, the $a_{rf}(t_{i})$ can be obtained through the following formula:

(9) $$a_{rf}(t_{i})=2(d_{n}(t_{i-1})+v_{p}(t_{i-1})\Delta t+a_{p}(t_{i}){\left(\Delta t\right)}^{2}/2-v_{f}(t_{i-1})\Delta t-L)/{\left(\Delta t\right)}^{2}$$

Here, L represents the desired distance headway between two e-bicycles in the stationary state.

### 3.3. Evaluation Indicators

To quantitatively assess the improvement of the AEB system in mitigating rear-end collision risks for e-bicycles during emergency braking events, this study selected a series of improved safety surrogate measures for evaluation. These indicators were used to measure the number of collisions, collision probability, and collision severity, respectively.

The number of rear-end collisions (NC) was adopted to visually represent the outcome after emergency braking. If a collision occurs when two e-bicycles are completely stationary, it is recorded as 1; otherwise, it is recorded as 0.

Based on the widely-used surrogate safety measure TTC, Minderhoud and Bovy [41] proposed the time-exposed time-to-collision (TET) and time-integrated time-to-collision (TIT) to further measure collision risks. These proposed indicators have been widely adopted and improved by researchers [42,43,44,45]. TET represents the time during which an e-bicycle is in a hazardous riding condition. TIT represents the cumulative collision risk value of an e-bicycle during hazardous riding conditions. In this study, the average value of TIT (ATIT) was further proposed to characterize the average collision risk value in hazardous riding conditions, which can indicate the collision probability of an e-bicycle during emergency braking. The mathematical expressions of the three indicators are as follows:

(10) $$TET=\sum_{t=t_{s}}^{t_{e}}\delta (t)\cdot \Delta t$$

(11) $$\delta (t)=\left\{ \begin{array}{l} 1,\;0\leq TTC\leq TTC_{thr}\\ 0,\;otherwise \end{array}\right.$$

(12) $$TIT=\int_{t_{s}}^{t_{e}}\left[TTC_{thr}-TTC(t)\right]\cdot d_{t},\;0\leq TTC(t)\leq TTC_{thr}$$

(13) ATIT=TIT/TET

Here, $t_{s}$ represents the start time of the emergency braking, $t_{e}$ represents the end time of the emergency braking, δ(t) represents a switching variable, Δt represents length of a time step, $TTC_{thr}$ represents the TTC threshold value. As mentioned earlier in this study, researchers often set the TTC threshold between 1 and 3 s, and the middle value of 2 s was adopted in this study when assessing collision probability.

Relative safety stop distance (RSD) is an extended measure based on the concept of safety stop distance that is used to assess the severity of collisions [42,46,47]. When the speed of the FEB is greater than the speed of the PEB, on the profile where RSD is less than 0, the absolute value of RSD increases. The larger the absolute value of RSD, the more severe the collision [42,47]. Based on the above concepts, to further characterize the collision severity during the emergency braking process, this study proposed the maximum value of RSD (MRSD) and the average value of RSD (ARSD). The mathematical expressions for these two indicators are as follows:

(14) $$RSD(t_{i})=SDP(t_{i})-SDF(t_{i})$$

(15) $$SDP(t_{i})=v_{p}(t_{i})\cdot h_{fp}(t_{i})+\frac{{\left(v_{p}(t_{i})\right)}^{2}}{2\cdot a}+l$$

(16) $$SDF(t_{i})=v_{f}(t_{i})\cdot p+\frac{{\left(v_{f}(t_{i})\right)}^{2}}{2\cdot a}$$

(17) $$MRSD=\max\left(\left|RSD(t_{i})\right|\right),RSD(t_{i})\leq 0\;t_{i}\in \left[t_{s},t_{e}\right]$$

(18) $$ARSD=\frac{\sum_{t=t_{s}}^{t_{e}}\left(\left|RSD(t_{i})\right|\right)}{T},RSD(t_{i})\leq 0\;t_{i}\in \left[t_{s},t_{e}\right]$$

Here, $SDP(t_{i})$ represents the safe stopping distance of the preceding e-bicycle at the time $t_{i}$, $SDF(t_{i})$ represents the safe stopping distance of the following e-bicycle at the time $t_{i}$, $v_{p}(t_{i})$ represents the speed of the preceding e-bicycle at the time $t_{i}$, $h_{fp}(t_{i})$ represents the time headway between the following e-bicycle and the preceding e-bicycle at the time $t_{i}$, a represents the expected deceleration rate, l represents the length of e-bicycle, $v_{f}(t_{i})$ represents the speed of the following e-bicycle at the time $t_{i}$, p represents the expected perception-reaction time, T represents the number of times that RSD is less than 0 within the period between $t_{s}$ and $t_{e}$.

### 3.4. The Scenario and Parameters Setting of Numerical Simulation

The simulation scenario of this study was a non-motorized lane in an urban area. The simulation time was set to 150 s with a time-step interval of 0.1 s. In each different riding condition, the numerical simulation was conducted a minimum of 30 times to ensure an adequate number of statistical samples. In order to avoid interference in the numerical simulation, the scenarios were designed without any vehicles or other types of non-motorized vehicles present. The scenario consists of two e-bicycles with an initial speed of 25 km/h (6.94 m/s) and an initial distance headway of 6 m. According to the safety technical specification for electric bicycles [48] in China, the maximum speed limit for an e-bicycle was set at 25 km/h. When the simulation ran for 100 s, the preceding e-bicycle performed an emergency braking with different deceleration rates (1.5 m/s^2^, 3.0 m/s^2^, and 4.5 m/s^2^) until its speed reached 0 km/h. In the condition without the AEB system, when the PEB brakes, the FEB iterates the speed and other variables at each time step based on the proposed e-bicycle following model. In the condition with the AEB system, the FEB performs state variable iteration in conjunction with the e-bicycle following the model and the AEB system process in Figure 2.

The comparative simulations were also conducted for the risk judgment criteria (TTC threshold) of the AEB system. The TTC threshold was set to three different values: 1 s, 2 s, and 3 s. In addition, the control of the AEB system may have system delays, such as communication delays and induction delays of the millimeter-wave radar. As a result, the delay time was set to 0 s, 0.1 s, and 0.2 s to simulate and analyze different control delay scenarios.

Overall, this study conducted a total of 1620 numerical simulations, including 810 simulations with the AEB system and 810 simulations without the AEB system. In the scenarios with the AEB system, the numerical simulations were designed in 3 (deceleration rates) × 3 (delay times) × 3 (TTC thresholds) scenarios, with each scenario conducting 30 simulations. In the scenarios without the AEB system, there were 3 (deceleration rates) scenarios, and to ensure that the data samples were matched for analysis, each scenario conducted 270 simulations. The construction and execution of the simulation scenario were performed in MATLAB R2018b software.

The parameters of the established e-bicycle following model were set based on previous research on the IDM model [38,49,50] and incorporated the behavioral characteristics of e-bicycles. Specifically, $a_{e}$ was set to 1 m/s^2^, $v_{\max}$ was set to 25 km/h, θ was set to 2.7, $\xi_{1}$ was set to 0.3 m/s^2^, $D_{1}$ was set to 4 m, $D_{2}$ was set to 8 m, $p_{1}$ was set to 0.15, Δt was set to 0.1 s, L was set to 1 m. In the parameter settings of the collision severity indicators, previous research was also referenced [42,46,51], and ultimately a was set to 3 m/s^2^, l d was set to 2.5 m, p was set to 1.5 s.

## 4. Results

The dependent variables under different riding conditions were analyzed to examine the effects of the AEB system, deceleration condition, delay time, and TTC threshold on the probability, severity, and occurrence of rear-end collisions. Analysis of variance (ANOVA) was adopted as the examination method. Chi-square tests were conducted to examine the relationship between independent variables and the NC. The hypothesis testing in all analyses was based on a significance level of 0.05. The statistical analysis mentioned above was conducted using SPSS 25.0 software.

Before the formal analysis, an ANOVA was used to investigate the impact of the independent variables on the average speed and distance headway of the e-bicycles in the following process, to avoid the occurrence of rear-end collisions due to significant differences in these behavior variables.

**Figure 4:**
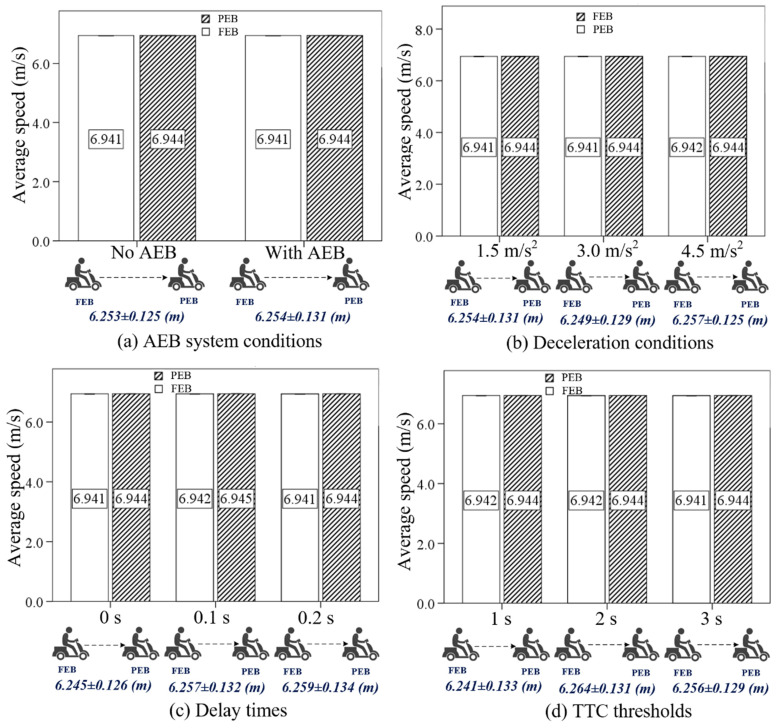
The results of average speed and distance headway under different riding conditions before emergency braking.

The descriptive statistics and the ANOVA results of the average speed and distance headway are listed in Figure 4 and Table 1. The results showed the AEB system, deceleration condition, delay time, and TTC threshold had no significant effects on the average speed and distance headway of e-bicycles before emergency braking. Figure 4 shows that in the different riding conditions, the average speed of the PEB and FEB and the distance headway between them were basically the same. The average speed was maintained at about 6.94 m/s, and the distance headway was maintained at about 6.25 m, which proves the effectiveness of the e-bicycle following model. Additionally, the results mean that the occurrence of rear-end collisions in this study was not caused by differences in average speed and distance headway before emergency braking.

### 4.1. Number of Rear-End Collisions

**Figure 5:**
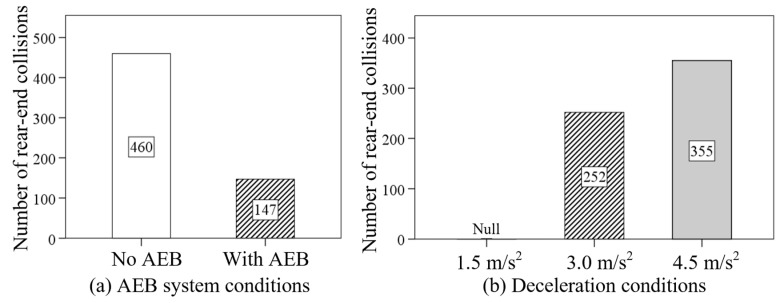
The effects of AEB system conditions and deceleration conditions on the NC.

Table 2 shows the descriptive statistical results of the number of rear-end collisions (NC) under different AEB system conditions and deceleration conditions, and Table 3 shows the Pearson chi-square results of the NC. The results showed significant effects of the AEB system (value = 258.110, *p* < 0.05) and the deceleration condition (value = 527.286, *p* < 0.05) on the NC. As depicted in Figure 5, the NC in the condition without the AEB system was significantly higher than the condition with the AEB. When the deceleration increased from 1.5 m/s^2^ to 4.5 m/s^2^, the NC increased from 0 to 355.

**Figure 6:**
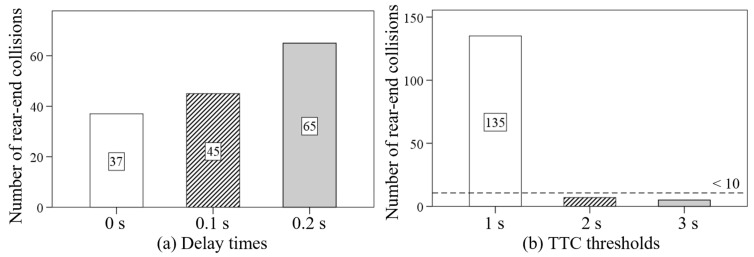
The effects of delay times and TTC thresholds on the NC.

The ANOVA results and descriptive statistics of the NC under different delay times and TTC thresholds are shown in Table 3 and Table 4. The delay time (value = 10.372, *p* < 0.05) and TTC thresholds (value = 276.657, *p* < 0.05) were found to have significant effects on the NC. Figure 6 shows that, as the delay time increased from 0 s to 0.2 s, the NC increased from 37 to 65. Conversely, as the TTC threshold increased from 1 s to 3 s, the NC dropped sharply from 135 to 5. Additionally, when the TTC threshold was set at 2 s and 3 s, the NC remained almost consistent.

### 4.2. Collision Probability

Table 5 lists the descriptive statistics of the time exposed time-to-collision (TET), time integrated time-to-collision (TIT), and the average value of time integrated time-to-collision (ATIT), and Table 6 shows the ANOVA results for the three variables. The results indicated significant effects of AEB system conditions on the TET (F = 90.730, *p* < 0.05), TIT (F = 279.908, *p* < 0.05), and ATIT (F = 543.117, *p* < 0.05). Similarly, the deceleration condition had significant effects on the TET (F = 421.221, *p* < 0.05), TIT (F = 694.873, *p* < 0.05), and ATIT (F = 1137.492, *p* < 0.05).

**Figure 7:**
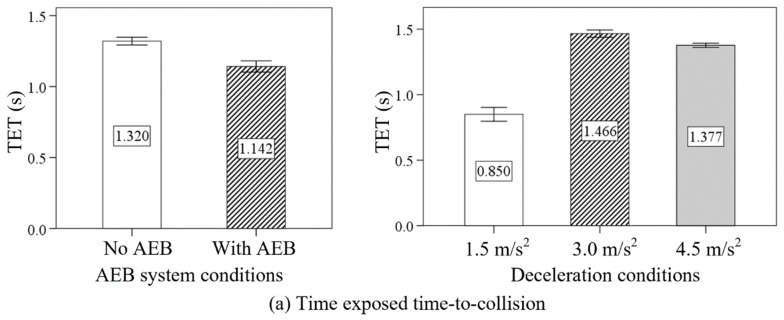

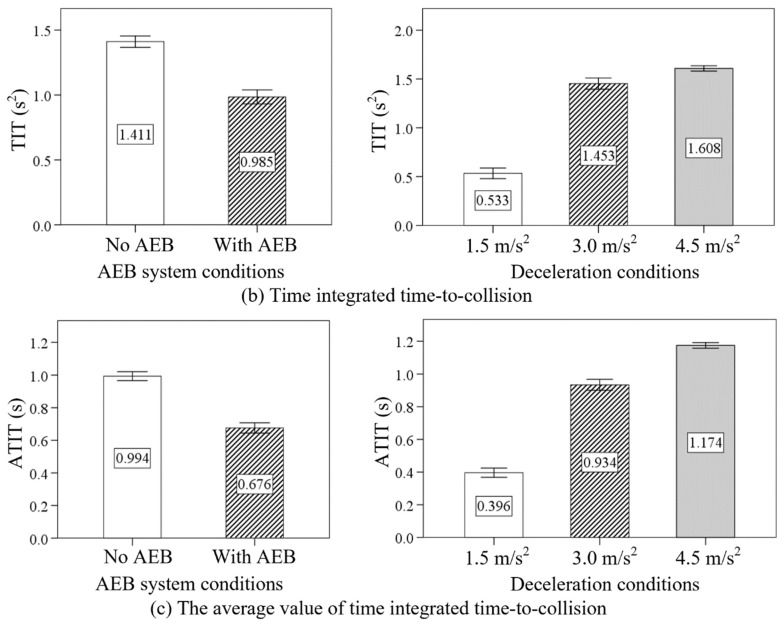
The effects of AEB system conditions and deceleration conditions on the TET, TIT, and ATIT.

The values of TET, TIT, and ATIT were all significantly smaller in the condition with AEB compared with those in no AEB condition. Moreover, Figure 7 shows that the values of TET were 0.850 s, 1.466 s, and 1.377 s, respectively, in low deceleration (1.5 m/s^2^), moderate deceleration (3.0 m/s^2^), and high deceleration (4.5 m/s^2^). In addition, the TIT and ATIT increased upward as the deceleration increased from 1.5 m/s^2^ to 4.5 m/s^2^.

The ANOVA results and descriptive statistics of the TET, TIT, and ATIT under different delay times and TTC thresholds are shown in Table 6 and Table 7. The delay time was found to have significant effects on the TET (F = 16.728, *p* < 0.05), TIT (F = 25.483, *p* < 0.05), and ATIT (F = 31.921, *p* < 0.05). There were also significant differences in TET (F = 23.705, *p* < 0.05), TIT (F = 93.394, *p* < 0.05), and ATIT (F = 126.294, *p* < 0.05) between low TTC threshold (1 s), moderate TTC threshold (2 s), and high TTC threshold (3 s) conditions.

**Figure 8:**
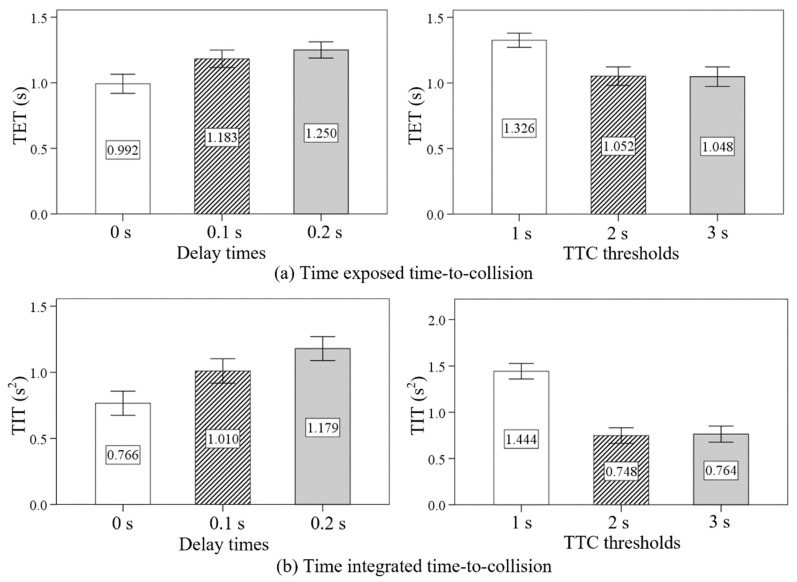

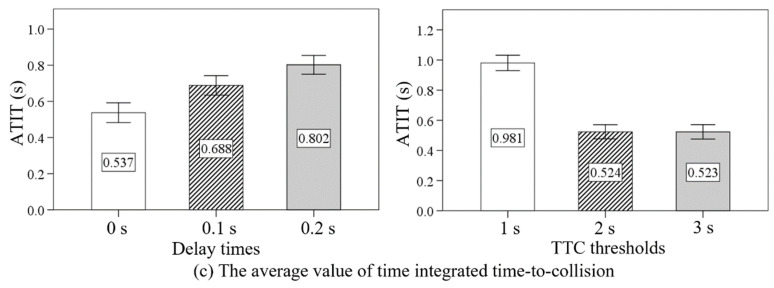
The effects of delay times and TTC thresholds on the TET, TIT, and ATIT.

As shown in Figure 8, an increasing tendency was observed on the TET, TIT, and ATIT as the delay time increased from no delay (0 s) to high delay (0.2 s). From the point of view of the TTC threshold, the values of TET, TIT, and ATIT were the largest in the low TTC threshold condition. However, the values of TET, TIT, and ATIT in the moderate TTC threshold condition and the high TTC threshold condition had no significant difference.

### 4.3. Collision Severity

Table 8 lists the descriptive statistics of the average value of relative safe stop distance (ARSD) and the maximum value of relative safe stop distance (MRSD), and Table 9 shows the ANOVA results for the ARSD and MRSD. The results indicated significant effects of AEB system conditions on the ARSD (F = 341.015, *p* < 0.05) and MRSD (F = 48.018, *p* < 0.05). The deceleration condition also had significant effects on the ARSD (F = 363.463, *p* < 0.05) and MRSD (F = 376.417, *p* < 0.05).

**Figure 9:**
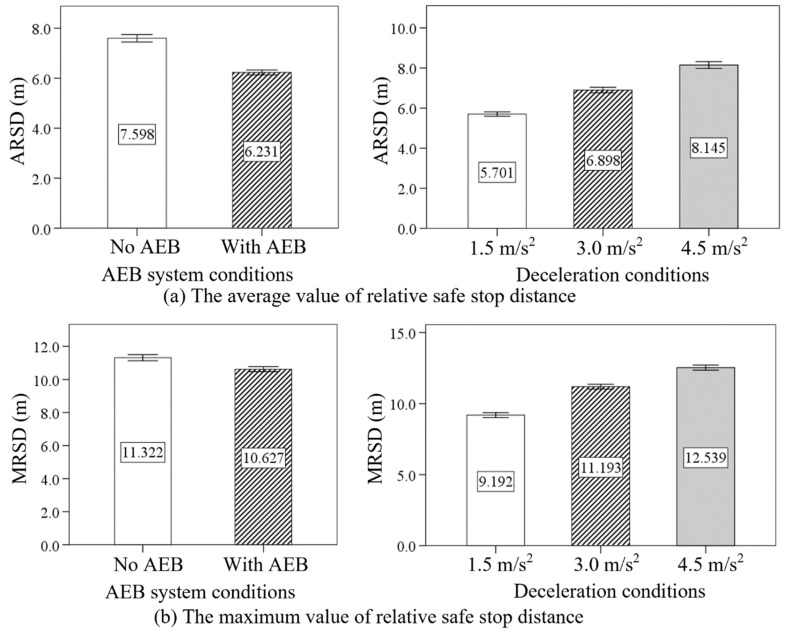
The effect of AEB system conditions and deceleration conditions on the ARSD and MRSD.

The values of ARSD and MRSD were all smaller in the condition with AEB compared with no AEB condition. In addition, Figure 9 shows the increasing tendency of the ARSD and MRSD as the deceleration increased from 1.5 m/s^2^ to 4.5 m/s^2^.

The ANOVA results and descriptive statistics of the ARSD and MRSD under different delay times and TTC thresholds are shown in Table 9 and Table 10. The delay time was found to have significant effects on the ARSD (F = 23.429, *p* < 0.05) and MRSD (F = 12.085, *p* < 0.05). There were also significant differences in ARSD (F = 91.802, *p* < 0.05) and MRSD (F = 21.785, *p* < 0.05) between low TTC threshold, moderate TTC threshold, and high TTC threshold conditions.

**Figure 10:**
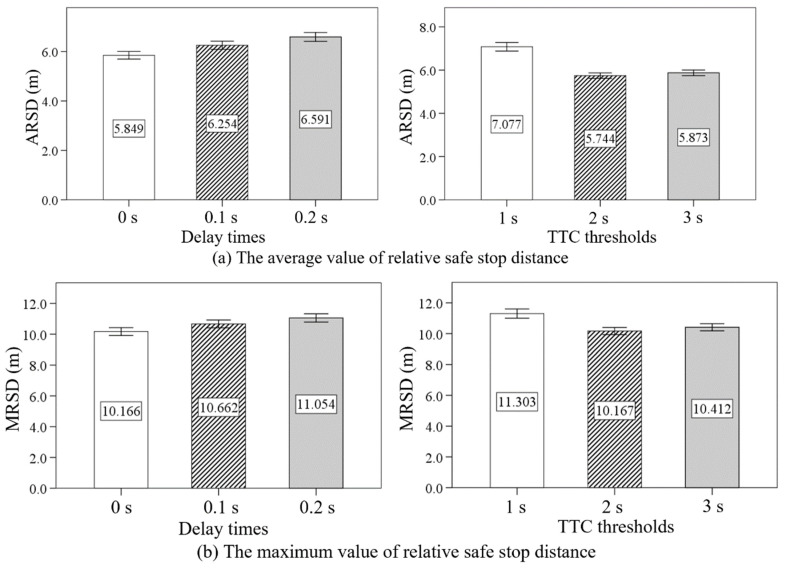
The effect of delay time and TTC threshold on the ARSD and MRSD.

As shown in Figure 10, an increasing tendency was observed on the ARSD and MRSD as the delay time increased from 0 s to 0.2 s. Moreover, the values of ARSD and MRSD were highest in the low TTC threshold condition. The values of ARSD and MRSD in the moderate TTC threshold condition were both slightly smaller than those in the high TTC threshold condition.

## 5. Discussion

E-bicycles are frequently involved in serious rear-end collisions on urban roads. Previous studies have proposed policy-oriented measures to prevent the occurrence of collisions but pose challenges in terms of effective enforcement. In this study, an autonomous emergency braking system (AEB) was designed, and an e-bicycle following model was proposed. Numerical simulation experiments were conducted in various riding conditions to examine the effect of the AEB. Based on the experiment results, the study systematically investigated the impact of factors such as deceleration conditions, the AEB system, system delay time, and the system TTC threshold on the risk of rear-end collisions involving e-bicycles.

Intense deceleration was a significant factor contributing to the increase in rear-end collisions involving e-bicycles. Experimental results indicated that no collisions occurred under the low deceleration condition (1.5 s/m^2^), while the number of rear-end collisions sharply increased under conditions of moderate (3.0 s/m^2^) to high deceleration (4.5 s/m^2^). Furthermore, the average severity of collisions occurring under moderate and high deceleration conditions increased by 21.0% and 42.9%, respectively, compared to the low deceleration condition. Therefore, intense deceleration is a hazardous riding behavior associated with a high risk of collision [52,53]. To enhance the safety of e-bicycle riders, it is crucial to reinforce safety awareness education to prevent hazardous riding behaviors that may trigger collisions [14,23].

The proposed AEB system effectively enhanced the safety of e-bicycle riders. In terms of the number of rear-end collisions, the condition with the AEB system resulted in a 68.0% reduction in collision numbers compared to the condition without the AEB system. The control effect of the proposed AEB system was similar to the impact of other active safety systems [15,21,54]. Specifically, under the condition of the AEB system, the collision probability for e-bicycles decreased by 32.0%, and the average severity of collision decreased by 18.0%. Therefore, the proposed active safety system effectively reduced the probability and severity of rear-end collisions for e-bicycles during emergency braking.

Based on the experimental results, it was observed that even with the AEB system, rear-end collisions involving e-bicycles were not entirely prevented. This highlights the significant impact of parameter settings in the AEB system on its active control effectiveness [24,25]. Specifically, when the TTC threshold was set at 1 s, there were 137 rear-end collisions, whereas for the other two TTC thresholds combined, there were only 12 collisions. This illustrates that a stringent TTC threshold provides minimal temporal and spatial margin for AEB system control, consequently resulting in collisions [27]. Furthermore, when the TTC thresholds were set at 2 s and 3 s, there was no significant difference in the number of rear-end collisions, collision probability, or collision severity between the two threshold conditions. As a result, the study recommends setting the TTC threshold at 2 s as the risk judgment criteria for the AEB system to avoid larger threshold values causing false alarms [35].

The control delay of the AEB system weakens its safety enhancement effect. Communication or physical delays in various systems reduce the system’s control function [55,56,57]. As the delay time of the AEB system increased from 0 s to 0.1 s and 0.2 s, the number of rear-end collisions increased by 21.6% and 75.7%, respectively. Additionally, the collision probability under a delay of 0.1 s and 0.2 s increased by 28.1% and 49.3% compared to no delay. The average severity of collisions at delay times of 0.1 s and 0.2 s also increased by 6.9% and 12.7% compared to no delay. However, it was found from the experimental results that when the AEB system has a delay time of 0 s or 0.1 s and the TTC threshold is set at 2 s or 3 s, the AEB system can completely prevent rear-end collisions.

Some limitations of this study should be noted. Firstly, in daily riding, e-bicycles often share the road with cars, and in future research, the characteristics of mixed traffic flow should be incorporated into the design of the AEB system and the improvement of the e-bicycle following model. Secondly, this study focused on rear-end collisions, while e-bicycles are also prone to right-angle collisions, so it is necessary to design an active safety system for this type of collision. Furthermore, it is worth investigating the higher requirements imposed on the AEB system and e-bicycle following model when the FEB is approaching the PEB with a higher speed. Finally, the experiment method of this study was the numerical simulation, and it should be noted that in the future, field data or real e-bicycle experiments should be used to enhance the generalizability of the e-bicycle following the model and the designed AEB system.

## 6. Conclusions

This study aimed to design an autonomous emergency braking (AEB) system to prevent rear-end collisions involving e-bicycles and investigated the impact of the AEB system, TTC thresholds, delay times, and deceleration conditions on the risk of rear-end collisions. Numerical simulation experiments were conducted using the proposed e-bicycle following model, and selected indicators were employed to perform a systematic risk assessment. The results clearly demonstrated that the proposed AEB system effectively reduces the number of rear-end collisions, decreases the collision probability, and mitigates collision severity. To achieve more effective prevention of rear-end collisions, it is crucial to establish appropriate system risk judgment criteria and ensure rapid system control. The study findings suggested that setting the TTC threshold at 2 s and limiting the system delay to below 0.1 s are optimal for maximizing the safety function of the AEB system. Additionally, the study identified intense deceleration as a significant contributing factor to rear-end collisions. This highlights the importance of reinforcing daily education for riders to avoid hazardous riding behaviors, such as aggressive braking, which can trigger collisions. The findings provided practical implications for the development of effective e-bicycle assistance technologies to promote safety. Moreover, the established e-bicycle following model and parameter settings could provide a good theory and algorithm for the e-bicycle microscopic simulation.

## Figures and Tables

**Table 1:** The ANOVA results of the average speed and headway distance.

Source	d.f.	F-Ratio		
		The Speed of PEB	The Speed of FEB	Distance Headway
AEB system conditions	1	0.031	0.003	0.007
Deceleration conditions	2	0.071	0.099	0.436
Delay times	2	0.971	0.808	0.906
TTC thresholds	2	0.087	0.272	2.256
AEB × Deceleration	2	0.449	0.558	2.669
Delay time × TTC threshold	4	1.803	1.835	0.152

**Table 2:** The descriptive statistics of NC under different AEB system conditions and deceleration conditions.

AEB System Condition	Deceleration Condition	NC
With AEB (N = 810)	−1.5 m/s^2^ (N = 270)	0
	−3.0 m/s^2^ (N = 270)	50
	−4.5 m/s^2^ (N = 270)	97
No AEB (N = 810)	−1.5 m/s^2^ (N = 270)	0
	−3.0 m/s^2^ (N = 270)	202
	−4.5 m/s^2^ (N = 270)	258

N represents the sample size.

**Table 3:** The Pearson Chi-Square results of the NC.

Source	Value	d.f.	Asymp.Sig. (2-Side d)
AEB system conditions	258.110	1	<0.001 *
Deceleration conditions	527.286	2	<0.001 *
Delay times	10.372	2	0.006 *
TTC thresholds	276.657	2	<0.001 *

* Significant at the 0.05 level.

**Table 4:** The descriptive statistics of NC under different delay times and TTC thresholds.

Delay Times	TTC Thresholds	NC
0 s (N = 270)	1 s (N = 90)	37
	2 s (N = 90)	0
	3 s (N = 90)	0
0.1 s (N = 270)	1 s (N = 90)	45
	2 s (N = 90)	0
	3 s (N = 90)	0
0.2 s (N = 270)	1 s (N = 90)	53
	2 s (N = 90)	7
	3 s (N = 90)	5

N represents the sample size.

**Table 5:** The descriptive statistics of TET, TIT, and ATIT under different AEB system conditions and deceleration conditions.

AEB Condition	Deceleration Condition	Parameter	TET (s)	TIT (s^2^)	ATIT (s)
With AEB (N = 810)	−1.5 m/s^2^ (N = 270)	Mean	0.549	0.254	0.253
		S.D.	0.499	0.405	0.243
	−3.0 m/s^2^(N = 270)	Mean	1.397	1.110	0.702
		S.D.	0.407	0.753	0.418
	−4.5 m/s^2^(N = 270)	Mean	1.479	1.591	1.073
		S.D.	0.180	0.416	0.245
No AEB (N = 810)	−1.5 m/s^2^(N = 270)	Mean	1.151	0.812	0.540
		S.D.	0.588	0.712	0.347
	−3.0 m/s^2^(N = 270)	Mean	1.534	1.796	1.166
		S.D.	0.201	0.360	0.189
	−4.5 m/s^2^(N = 270)	Mean	1.274	1.625	1.275
		S.D.	0.138	0.182	0.046

N represents the sample size.

**Table 6:** The ANOVA results of the TET, TIT, and ATIT.

Source	d.f.	F-Ratio		
		TET	TIT	ATIT
AEB system conditions	1	90.730 *	279.908 *	543.117 *
Deceleration conditions	2	421.221 *	694.873 *	1137.492 *
Delay times	2	16.728 *	25.483 *	31.921 *
TTC thresholds	2	23.705 *	93.394 *	126.294 *
Warning × Deceleration	2	156.239 *	61.380 *	31.965 *
Delay time × TTC threshold	4	7.877 *	9.166 *	6.010 *

* Significant at the 0.05 level.

**Table 7:** The descriptive statistics of TET, TIT, and ATIT under different delay times and TTC thresholds.

Delay Times	TTC Thresholds	Parameter	TET (s)	TIT (s^2^)	ATIT (s)
0 s (N = 270)	1 s (N = 90)	Mean	1.370	1.465	0.954
		S.D.	0.463	0.749	0.433
	2 s (N = 90)	Mean	0.836	0.429	0.339
		S.D.	0.579	0.474	0.293
	3 s (N = 90)	Mean	0.771	0.404	0.319
		S.D.	0.588	0.480	0.302
0.1 s (N = 270)	1 s (N = 90)	Mean	1.336	1.483	0.999
		S.D.	0.459	0.698	0.428
	2 s (N = 90)	Mean	1.080	0.746	0.525
		S.D.	0.561	0.686	0.378
	3 s (N = 90)	Mean	1.133	0.803	0.541
		S.D.	0.609	0.700	0.371
0.2 s (N = 270)	1 s (N = 90)	Mean	1.271	1.383	0.989
		S.D.	0.429	0.641	0.419
	2 s (N = 90)	Mean	1.240	1.069	0.707
		S.D.	0.548	0.768	0.407
	3 s (N = 90)	Mean	1.239	1.084	0.710
		S.D.	0.568	0.800	0.415

N represents the sample size.

**Table 8:** The descriptive statistics of ARSD and MRSD under different AEB system conditions and deceleration conditions.

AEB Condition	Deceleration Condition	Parameter	ARSD (m)	MRSD (m)
With AEB (N = 810)	−1.5 m/s^2^ (N = 270)	Mean	5.469	9.130
		S.D.	1.135	1.911
	−3.0 m/s^2^ (N = 270)	Mean	6.194	10.886
		S.D.	1.242	1.860
	−4.5 m/s^2^ (N = 270)	Mean	7.031	11.866
		S.D.	1.436	1.862
No AEB (N = 810)	−1.5 m/s^2^ (N = 270)	Mean	5.933	9.253
		S.D.	1.390	2.202
	−3.0 m/s^2^ (N = 270)	Mean	7.602	11.501
		S.D.	1.671	2.109
	−4.5 m/s^2^ (N = 270)	Mean	9.259	13.212
		S.D.	1.923	2.131

N represents the sample size.

**Table 9:** The ANOVA results of the ARSD and MRSD.

Source	d.f.	F-Ratio	
		ARSD	MRSD
AEB system conditions	1	341.015 *	48.018 *
Deceleration conditions	2	363.463 *	376.417 *
Delay times	2	23.429 *	12.085 *
TTC thresholds	2	91.802 *	21.785 *
Warning × Deceleration	2	47.355 *	12.560 *
Delay time × TTC threshold	4	0.929	2.295

* Significant at the 0.05 level.

**Table 10:** The descriptive statistics of ARSD and MRSD under different delay times and TTC thresholds.

Delay Times	TTC Thresholds	Parameter	ARSD (m)	MRSD (m)
0 s (N = 270)	1 s (N = 90)	Mean	6.842	11.243
		S.D.	1.451	2.559
	2 s (N = 90)	Mean	5.358	9.665
		S.D.	0.767	1.520
	3 s (N = 90)	Mean	5.347	9.591
		S.D.	0.913	1.727
0.1 s (N = 270)	1 s (N = 90)	Mean	7.022	11.130
		S.D.	1.676	2.464
	2 s (N = 90)	Mean	5.793	10.280
		S.D.	1.051	1.905
	3 s (N = 90)	Mean	5.946	10.576
		S.D.	1.006	1.883
0.2 s (N = 270)	1 s (N = 90)	Mean	7.367	11.534
		S.D.	1.799	2.444
	2 s (N = 90)	Mean	6.081	10.558
		S.D.	1.184	2.246
	3 s (N = 90)	Mean	6.325	11.070
		S.D.	1.104	1.922

N represents the sample size.

## Data Availability

Data are available on request due to restrictions. The data presented in this study are available on request from the corresponding author. The data are not publicly available due to data protection or privacy concerns.
